# Chylothorax bilatéral au cours de la maladie de Behçet

**DOI:** 10.11604/pamj.2015.20.370.5316

**Published:** 2015-04-15

**Authors:** Naaima Zemed, Nadia Amangar, Laila Herrak, Mustapha Elftouh

**Affiliations:** 1 Service de Pneumologie de l'Hôpital Ibn Sina, CHU Rabat, Maroc

**Keywords:** Maladie de Behçet, chylothorax, thrombose, Behçet disease, chylothorax, thrombosis

## Abstract

L'atteinte médiastino-pulmonaire de la maladie de Behçet est représentée essentiellement par la thrombose de la veine cave supérieure et l'angéite pulmonaire. L'association de la maladie et du chylothorax est rare, sa prise en charge n'est pas codifiée. Nous rapportant un cas clinique concernant la survenue d'un chylothorax bilatéral secondaire à une thrombose cave supérieure dans un contexte de maladie de Behçet.

## Introduction

La maladie de Behçet est une vascularite multisystémique à tropisme veineux, caractérisée par la survenue de poussées successives entrecoupées de rémissions avec un polymorphisme clinique et évolutif allant des formes bénignes cutanéomuqueuses et articulaires aux formes sévères, mettant en jeu le pronostic fonctionnel et/ou vital à savoir, l'atteinte oculaire, vasculaire et neurologique. L'atteinte médiastino-pulmonaire est rare mais grave, représentée essentiellement par la thrombose de la veine cave supérieure et l'angéite pulmonaire. La survenue d´un chylothorax au cours de la maladie est rare.

## Patient et observation

Nous rapportons l´observation médicale d´un patient âgé de 43 ans, marocain, agriculteur de profession, ancien tabagique chronique à raison de 16 paquet-année, suivi depuis trois mois pour maladie de Behçet retenue sur la présence d´aphtose buccale récurrente (6 épisodes au cours de la dernière année), des pseudofolliculites, d´une atteinte vasculaire à type de thrombose de la veine cave supérieure, et d´une uvéite postérieure. Le patient est mis sous traitement anticoagulant (héparinothérapie à does curative relayée par les anitivitamine K), corticothérapie orale (1 mg/kg par jour) et bolus mensuel de cyclophosphamide (15 mg/kg par mois).

Par ailleurs, il se plaignait d'une dyspnée stade III de la NYHA apparue depuis 4 semaines, sans autres signes associés, dans un contexte de conservation de l´état général. A l'examen physique, il était apyrétique, sa saturation en oxygène était de 96% à l´air ambiant, sa fréquence respiratoire de 16 cycles par minute, son rythme cardiaque de 96 battements par minute, sa tension artérielle de 120/60 mmHg. L'examen somatique a objectivé un syndrome d´épanchement liquidien des deux tiers inférieurs de l'hémothorax de façon bilatérale, une dermite ocre des deux membres inférieurs, et une pseudofolliculite étendue du visage et du dos. Les bruits du coeur étaient bien perçus et la sonorité abdominale était conservée. La radiographie du thorax a révélé un épanchement pleural bilatéral sans cardiomégalie. La tomodensitométrie thoracique a confirmé la présence d'un épanchement pleural bilatéral prédominant à droite avec une importante circulation médiastinale collatérale. La ponction pleurale a ramené du liquide blanc lactescent, dont l'analyse chimique et cytolobactériologique a montré une cellularité lymphocytaire aseptique avec une réaction de Rivalta positive et un taux de triglycérides à 4,25 g/L, avec présence de chylomicrons. Le bilan biologique a montré un taux de globules blancs à 6280/mm3, une hémoglobine à 12,8 g/dl et un taux des plaquettes à 226 000/mm3. La protidémie était normale à 68g/l. Le bilan lipidique sérique était sans anomalie notable. la fonction rénale et hépatique étaient normales. Nous avons réalisé une IRM thoracoabdominale à la recherche d'un obstacle sur le trajet du canal thoracique qui est revenu sans particularité. Le patient ne présentait aucune cause traumatique, néoplasique ou chirurgicale qui pouvait expliquer la présence de chylothorax.

Nous avons retenu le diagnostic de chylothorax bilatéral secondaire à une thrombose cave supérieure dans un contexte de maladie de Behçet. La prise en charge thérapeutique consiste en un drainage thoracique bilatéral (à droite puis à gauche avec un intervalle de 24h) associé à un régime pauvre en graisse. Le traitement par cyclophosphamide et par anticoagulant a été poursuivi. L’évolution était marqué par le tarissement de l’épanchement motivant le retrait des drains (à J 21 du drainage a droite et à J 19 à gauche). A six mois d’évolution de patient a présenté une nette régression du syndrome cave supérieure et à une année il ne présente pas de récidive.

## Discussion

La maladie de Behçet est une vascularite systémique dont la pathogénie est encore discutée. Elle fait intervenir plusieurs facteurs: génétiques, infectieux et immunitaires [1]. Le substratum anatomique de cette maladie est une vascularite capable de toucher tous les vaisseaux, quels que soient leur nature et leur calibre, avec néanmoins une prédominance pour la localisation veineuse qui recouvre 80 à 90% des atteintes vasculaires, elle est en moyenne observée chez un tiers des patients [2]. L'atteinte est ubiquitaire mais prédomine nettement autour du bassin méditerranéen, au Moyen Orient et au Japon [3]. Elle touche surtout l'homme jeune associant une aphtose buccogénitale à des manifestations systémiques diverses. Le diagnostic est clinique, reposant sur des regroupements symptomatiques. La diversité des critères de diagnostic propres à chaque pays ou à chaque école a conduit en 1990 à la publication des critères de classification dits internationaux qui on une sensibilité de 97% et une spécificité de 96% [4].

Parmi ses manifestations multiples et variées, l'atteinte pulmonaire est classiquement rare et de mauvais pronostic [5]. Précédées par la Les thromboses veineuses profondes des membres inférieurs, les thromboses caves correspondent à la deuxième localisation de l'atteinte veineuse en termes de fréquence et regroupent près d'un quart des cas des manifestations thrombotiques [2]. La thrombose cave supérieure est rapportée dans 2,5% des cas [5] constitue le mécanisme le plus probable des collections chyleuses au cours de la maladie en provoquant l'obstruction de l'orifice du canal thoracique, ce qui conduit à une augmentation de la pression intraluminale, une pression en retour dans les vaisseaux communicants et à la fuite du chyle depuis les canaux lymphatiques dans la cavité pleurale [6]. La pathogénie de l'association de la maladie de Behçet et du chylothorax reste incertaine. Dans la littérature, seulement quelques cas ont été rapportés sur la survenue de l’épanchement chyleux avec une thrombose cave supérieure au cours de la maladie. Le chylopéricarde et l'ascite chyleuse peuvent également se voir [7, 8].

Selon les recommandations de l'EULAR publiés en 2008, tout patient ayant une uvéite postérieure doit bénéficier en première intention d'une association corticoïdes et azathioprine et l'atteinte vasculaire veineuse ou artérielle justifie le recours systématique aux immunosuppresseurs [9]. La prise en charge du chylothorax est basée essentiellement sur le traitement de la thrombose basé sur les anticoagulants, le régime pauvre en graisse voir l'interruption de l'alimentation orale et la mise en place d'une nutrition entièrement parentérale, pouvant aider à stopper l'apport digestif en triglycérides et ainsi à ralentir la constitution de la chyle. Le drainage thoracique ou dans certain cas la pleurodèse ont été à l'origine d'une évolution favorable [10]. A notre connaissance, la ligature du canal thoracique n'a pas été expérimentée chez ces patients.

## Conclusion

La survenue d'un chylothorax au cours de la maladie de Behçet, sans cause traumatique néoplasique ou chirurgicale, est rare et doit faire rechercher une thrombose de la veine cave supérieure qui constitue le mécanisme le plus probable des collections chyleuses au cours de la maladie. La prise en charge n'est pas codifiée et repose essentiellement sur le traitement de la thrombose et les mesures diététiques. Un drainage thoracique ou une pleurodèse peuvent être proposés.
